# Long term surgical results of cases with aneurysmal bone cysts—a comprehensive clinical, functional and oncologic analysis

**DOI:** 10.1186/s12891-025-09109-6

**Published:** 2025-10-23

**Authors:** Okan Yigit, Emrecan Akgun, Emrah Gokay Ozgur, Omer Sofulu, Servet Igrek, Evrim Sirin, Bulent Erol

**Affiliations:** 1 Department of Orthopaedics and Traumatology, Kartal Dr. Lutfi Kirdar City Hospital, Istanbul, 34865 Turkey; 2 Department of Orthopaedics and Traumatology, Sultanbeyli State Hospital, Istanbul, 34935 Turkey; 3 Department of Biostatistics, Marmara University, Istanbul, 34854 Turkey; 4 Department of Orthopaedics and Traumatology, Pendik Training and Research Hospital, Istanbul, 34899 Turkey

**Keywords:** Aneurysmal bone cyst, Long-term results, Treatment

## Abstract

**Background:**

This study aims to analyze long-term outcomes in patients with aneurysmal bone cysts (ABCs) and demonstrate effective disease control. In the literature, the absence of a long-term study systematically evaluating the outcomes of a population with such a significant number of patients from different age groups will shed light on our surgical preferences.

**Methods:**

The study encompassed ABC diagnoses, primarily concerning long bones (46 women, 62 men; mean age 19.4 years; range 2—67) extracted from patient records spanning from 2007 to 2022 in our clinic. The average follow-up was 81 months. Extended intralesional curettage was performed using a mechanical burr, cauterization, grafting and internal fixation in 96 patients (88.9%), whereas resection was carried out in 12 patients (11.1%).

**Results:**

Pathological fractures occurred in 35% of cases. Reconstruction was performed in five patients (4.6%; endoprosthetic in 4, biological reconstruction in 1) and seven patients (6.5%) required no reconstruction. The mean Musculoskeletal Tumor Society (MSTS) score was 95.5 (range 60–100). Complications and recurrence rates were 12% and 9.3%, respectively. Surgical choice and adjuvant agent significantly influenced long-term complications and recurrence (*p* = 0.001).

**Conclusions:**

In the treatment management of aneurysmal bone cysts, extended intralesional curettage surgery coupled with the use of electrocautery and a high-speed burr has shown successful outcomes in long-term follow-up. A wider sample size with prospective randomized studies can be conducted regarding the treatment of ABCs.

## Introduction

Patients diagnosed with aneurysmal bone cysts (ABCs) were initially discussed in a study on simple bone cysts, with the terminology shaping the understanding of morphological images through histopathological evaluation [1]. Coined from observed vascular hemodynamic changes, the terms'aneurysm'and'cyst'originated from trauma-induced cyst development with an arteriovenous fistula [2, 3]. While generally considered benign, ABCs can exhibit local aggressiveness. A 2020 World Health Organization (WHO) classification places ABCs in a tumor group characterized by osteoclastic giant cells [4].

ABCs can be diagnosed using advanced histopathological tools such as FISH (Fluorescence In Situ Hybridization), next-generation sequencing, and USP6 gene detection [5]. Preoperative biopsy is essential to rule out differential diagnoses, including telangiectatic osteosarcoma and giant cell tumor of bone. Treatment options for ABCs include extended intralesional curettage, en-bloc resection and instillation therapy, each with distinct advantages and limitations [6, 7]. Curettage preserves bone but carries a higher recurrence risk, while resection minimizes recurrence but may necessitate reconstruction. Instillation therapy is minimally invasive but may be less effective for extensive lesions. Post-resection, reconstruction options include biological grafts or endoprostheses, and after extended curettage, medullary defects can be addressed with bone grafts or cement [8–10]. The treatment approach should be tailored to the lesion’s characteristics and patient needs.

The aim of this work was to retrospectively evaluate the long-term clinical, radiological, functional and oncological outcomes of cases with long bone and pelvic aneurysmal bone cysts presented at our clinic in the period from 2007 to 2022, along with complication rates in orthopedic patients.

## Materials and methods

In our retrospective single center study, we analyzed 108 patients diagnosed with ABCs at our hospital from 2007 to 2022. Primary data sources included outpatient records, pathology reports, surgery notes and orthopedic oncology records. Radiological assessments and functional evaluations utilized the hospital's imaging system and Musculoskeletal Tumor Society (MSTS) scoring. Exclusions comprised patients with follow-up period of fewer than 12 months, irregular records and secondary ABCs or other benign bone tumors. Ethical approval was obtained (Approval No. 09.2023.263).

We analyzed patient demographics including age and gender, duration of complaints, tumor localization, presence of fractures, imaging findings, disease stage, histopathological characteristics, surgical interventions, previous treatment history, clinical assessments, radiological evaluations, functional outcomes, oncological results, complication rates and recurrence status. Among 108 patients, nine patients (8%) had prior external treatment with 99 (92%) initiating care at our clinic. Curettage was chosen for those with prior treatment and persistent complaints. Initial complaints included regional pain, limb swelling and restricted movement leading to mobility issues.

All patients underwent conventional radiography (anteroposterior/lateral) and magnetic resonance imaging (MRI) for evaluation. Computed tomography (CT) and bone scintigraphy were conducted for pelvic, clavicle, and most long bone involvement (82 patients). The 3-dimensional capabilities of CT aided surgical planning. Using CT and MRI examinations, we scrutinized the lesion's precise location relative to anatomical structures, its adjacency to the cortex, proximity to the growth plate, and its borders with adjacent bone tissue. Scintigraphy is utilized to detect additional associated or independent lesions and offers a comprehensive view of the entire compartment, thereby facilitating whole-body scanning.

**Fig. 1:**
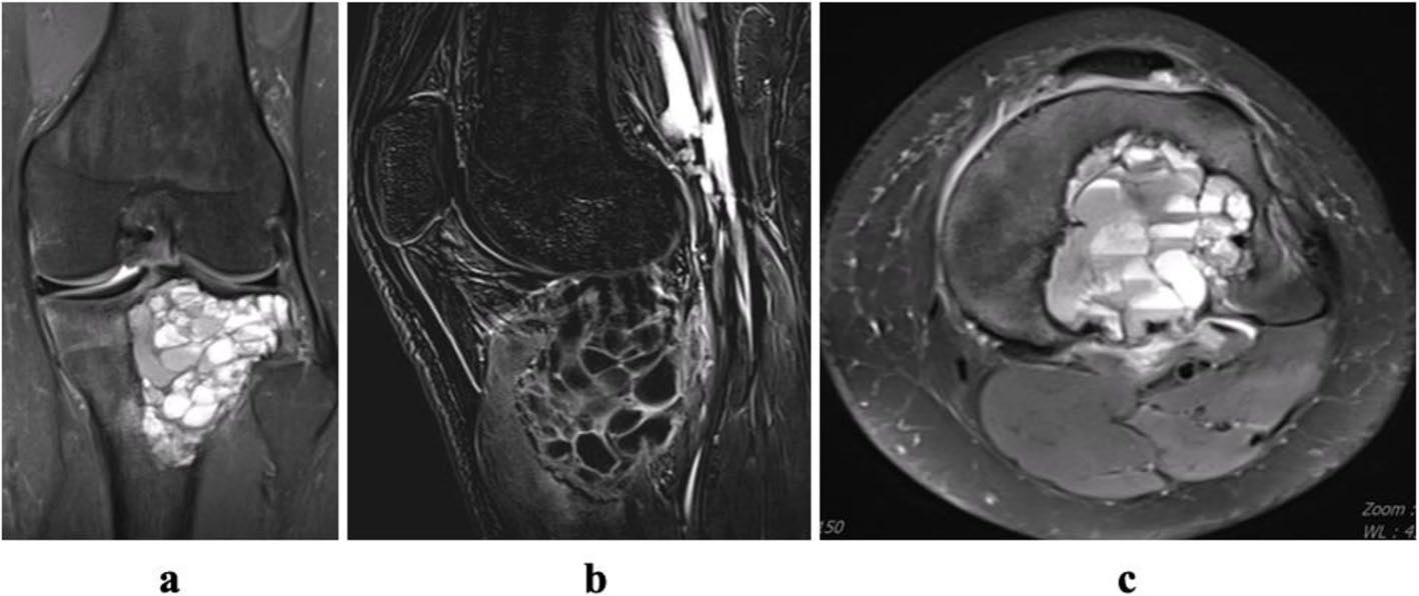
**a** Coronal MRI image showing fluid–fluid level. **b** Sagittal MRI image showing septa formation. **c** Axial MRI image showing the level difference

Orthopedic oncology performed radiographic examination and staging. Cyst volume, calculated via MRI, used digital coronal and sagittal images. Formulas for cylindrical (ABC × 0.785 (π × A/2 × B/2 × C)) and spherical (ABC × 0.52 (4/3 × π × A/2 × B/2 × C/2)) lesions were applied [11]. Campanacci classification determined radiological staging [12]. Inactive cysts showed a periosteal shell; active cysts lacked it, surrounded by bone; aggressive cysts had unclear boundaries. MRI section depicted fluid–fluid level and septa formation in a proximal tibia case (Fig. 1).

Patients were categorized by bone location (proximal, diaphyseal, distal) and involvement in long, short, flat or irregular bones. The long bones encompassed the femur, tibia, humerus, radius, ulna, metacarpals, metatarsals and phalanges. The short bones comprised the carpal and tarsal bones, as well as the patella. Flat bones encompassed the pelvis and scapula. Irregular bones include the sacrum and coccyx. Pathological fractures included micro-displacement or displacement cases. Follow-up assessed bone fusion, graft hypertrophy and osteointegration, evaluated by the orthopedic oncology team using X-rays.

At the 12-month post-surgery follow-up, clinical and functional evaluations were conducted. For upper extremities, factors like pain, function, emotional acceptance, hand position, skill and lifting ability were considered. Lower extremities were assessed for pain, function, emotional acceptance, need for walking support, walking status and gait style. Recurrence assessment for aneurysmal bone cysts involved imaging (X-rays, MRI, CT) and clinical examinations, noting localized swelling, tenderness and movement restrictions.

Follow-ups were biweekly for the initial three months, adjusted to three months for the first year, semi-annually in the second year and annually thereafter. Surgical site infections were monitored and suspected recurrence cases underwent advanced imaging. When evaluated alongside clinical and radiological findings, frozen biopsy was performed in certain patients where aneurysmal bone cyst was highly suspected, allowing the exclusion of malignancy and proceeding with surgical intervention. In other patients, the first stage involved confirming the diagnosis through core needle biopsy, followed by a surgical procedure to complete the second stage. Histopathological evidence confirming the diagnosis of aneurysmal bone cysts was obtained for each patient included in the study. Pathological evaluations based on core needle and frozen biopsies were reviewed in weekly oncology meetings to formulate surgical plans. In cases of recurrence, particularly those associated with inadequate curettage and irregular follow-up, treatment primarily involved resection, with extended curettage surgeries also being planned as an alternative approach.

### Surgical planning and technique

Surgical treatment options were typically determined by the oncology council based on factors such as the size and localization of the cyst within the bone, the severity of symptoms, and the expected quality of life for the patient. Extended curettage was generally recommended for large cysts, pathologic fractures or cysts that cause limb deformities. Reconstruction options were considered for recurrent cases or patient groups with irregular follow-up. The selection of these methods were guided by the individual patient's clinical condition and radiological findings, with detailed evaluation of surgical indications conducted within our oncology council.

The same surgical team performed all procedures. Extended intralesional curettage was done on 96 patients, while resection was chosen for 12. Reconstruction involved endoprosthetic or biological methods in five patients (5%) and seven patients (6.5%) required no reconstruction. Resection sites included sacrum (1%), fibula (4%) and clavicle (2%). Five patients with aggressive ABCs underwent wide resection followed by endoprosthetic or biological reconstruction on the femur (distal femur—3, proximal femur—1) and tibial diaphysis.

**Fig. 2:**
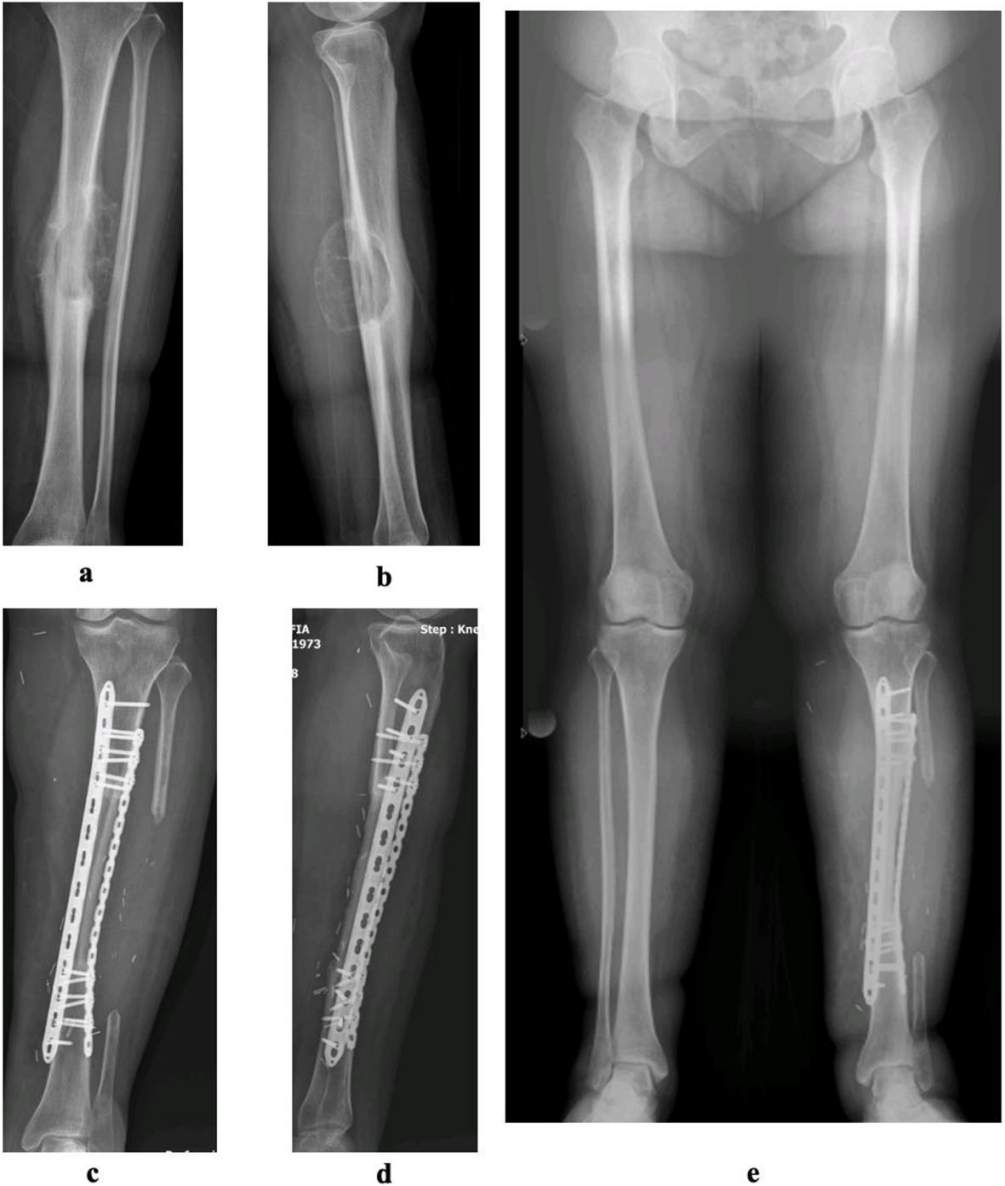
**a** Preoperative AP image of ABC in the diaphyseal tibial region. **b** Lateral plan view. **c** AP image of a patient who underwent reconstruction with vascularized fibula in the second postoperative year. **d** Postoperative lateral plan view. **e** Leg length radiography at eighth postoperative year

In the curettage group, grafts were used in 65 patients (60%), bone cement in 17 (16%) and no adjuvant agents in 14 (13%). Internal fixation utilized various implants (Kirschner wire, plates, screws, intramedullary nails) in 33 patients (34%). Control images for early and long-term follow-up of patients with biological reconstruction are provided (Fig. 2).

In cases with intraoperative frozen biopsies, tissue examination confirmed the absence of malignant potential. Single-stage surgery was completed with tru-cut biopsy samples obtained in the operating room under scopical device guidance. Following evaluation, the final surgery was planned and executed.

**Fig. 3:**
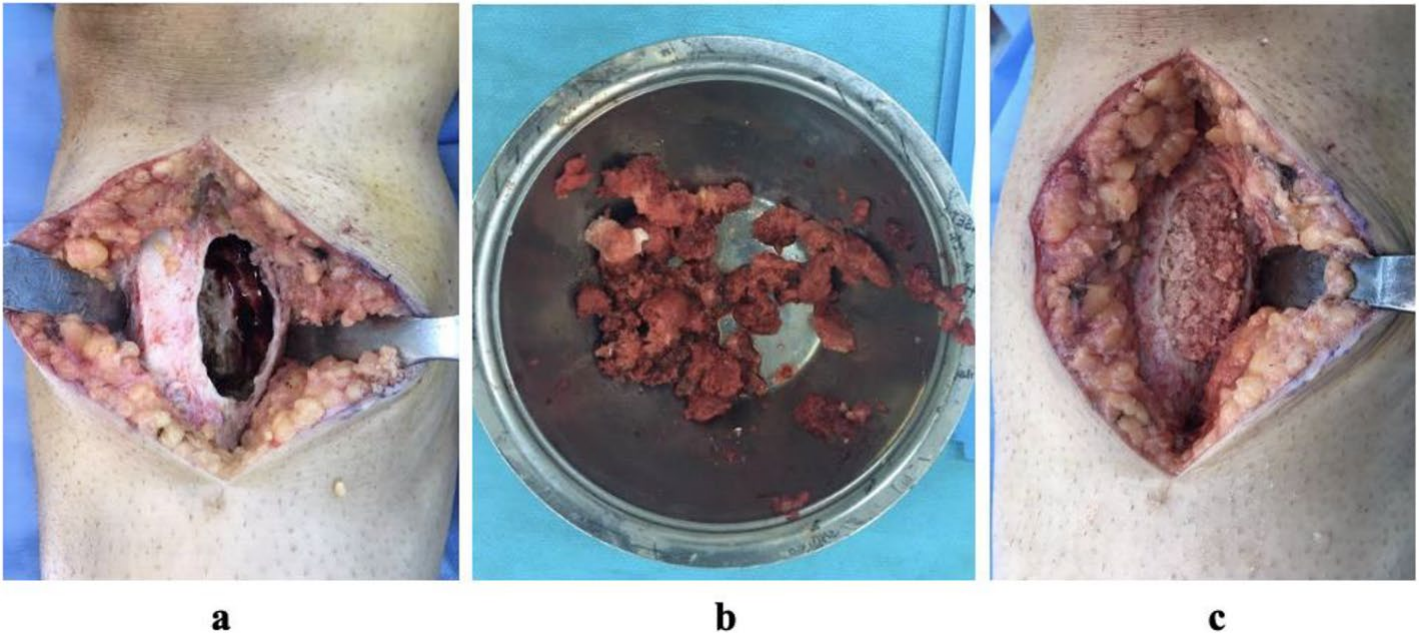
**a** An image of the opening of an oval-shaped lid on the bone cortex and then the extended curettage process. **b** An image of the material removed after curettage. **c** Filling of the medulla with graft support for the resulting bone defect

Initially, the surgery identified the region with the weakest bone cortex density. An oval-shaped cortical section was removed (Fig. 3) and samples were collected for preliminary diagnosis from bone tissue or hemorrhagic fluid material. The oval-shaped window was expanded, and the lesion was thoroughly curetted. The cyst walls were meticulously cleaned using a high-speed burr and electrocautery with multiple repetitions. Special attention was given to safeguard the epiphyseal plate, particularly in children and adolescents with an open physis. In cases extending beneath the physis, excision was achieved exclusively through curettage, guided by fluoroscopy. Macroscopic examination involved visualizing the physeal line under fluoroscopy and affected areas of the growth plate underwent multiple curettage iterations.

The fibulas were harvested using a lateral approach, ensuring retention of at least the distal 8 cm length in all patients to maintain ankle stability. Following dissection, the neurovascular bundle was identified in the distal 3 cm of the metaphyseal region of the fibula neck and prepared for anastomosis. In all instances, the fibula grafts were affixed to the bone as an adjunct segment for reconstruction. Plate and screws were employed for stabilization in specific cases of reconstruction.

Microscopic examination during the curettage procedure was accomplished by visualizing the growth plate line through a surgical loupe, creating a safe surgical margin behind the reactive zone of the lesion. Thorough washing of the surgical site with pulsatile lavage followed the procedure to remove debris tissues.

Local adjuvant agents like liquid nitrogen, phenol or alcohol were not used in any of these procedures. In cases with displaced pathological fractures, an initial reduction procedure was performed before continuing the surgical process. The electrocautery device was used in a blended mode, combining cutting and coagulation. It was occasionally switched to coagulation mode for hemostasis. Speed adjustment when using a burr was crucial for controlled cutting efficiency, preventing unnecessary damage to surrounding tissues.

Various materials, including allograft, autograft and bone cement, were employed to fill the resected bone gap. Autografts from the iliac and fibular bones were preferred, especially in cases involving children and adolescents with open physes. Bone grafts were primarily used in these cases to avoid potential thermal effects on the growth plate and subchondral bone associated with bone cement. On the other hand, bone cement was the preferred option in cases with closed physes in adolescents, aggressive lesions, recurrent diseases and adult patients.

Patient data were recorded and analyzed thoroughly with detailed demographic characteristics presented in Table 1. Statistical analysis was performed using the SPSS 20.0 software program (IBM, Armonk, NY, USA). Informed consent signatures were obtained from all study participants during the preoperative period and information about the follow-up period was provided.

**Table 1:** Demographic characteristics

Variables	n (%)
Gender	
Female	46 (42.6)
Male	62 (57.4)
Distribution of Patients	
Child	65 (60.2)
Adult	43 (39.8)
Age	
Child	11.3 ± 4.0
Adult	31.7 ± 13.1
Total	19.4 ± 13.3
Volume	96,294.4 ± 194,412.7
MSTS	95.5 ± 7.2
Follow-Up Period (month)	81 ± 58.2

Values are the means ± standard deviations

### Statistical analysis

Statistical analyses were performed using IBM SPSS 20.0 software (IBM Corp., Armonk, NY, USA). The normality of data distribution was assessed using the Kolmogorov–Smirnov test. Continuous variables are presented as median (Q1-Q3) due to non-normal distributions, and categorical variables are expressed as frequency (percentage). Comparisons between two groups were made using the Mann–Whitney U test for continuous variables and the Chi-square test for categorical variables. For comparisons involving more than two groups, the Kruskal–Wallis test was used. A *p*-value of < 0.05 was considered statistically significant for all analyses. The results of the univariate analysis were compared with those of the multivariate model, with risk assessment expressed as odds ratios (e.g., a'1.1-fold increase'and a'3.61-fold decrease'). The study power was calculated using G*Power software. For the Chi-square test, a sample size of 108 patients achieved 99% power to detect an effect size (W) of 0.5651, using 10 degrees of freedom with a significance level (alpha) of 0.05. This indicates a high probability of identifying significant differences if they exist.

## Results

Among 108 patients, 46 (42.6%) were women and 62 (57.4%) were men. Of these, 65 (60.2%) were children and 43 (39.8%) were adults. The majority (84.2%) were in the first three decades of life. Average MSTS score was 95.5, average age 19.4 years, average follow-up 81 months and average mass volume 96.2 cm^3^.

**Fig. 4:**
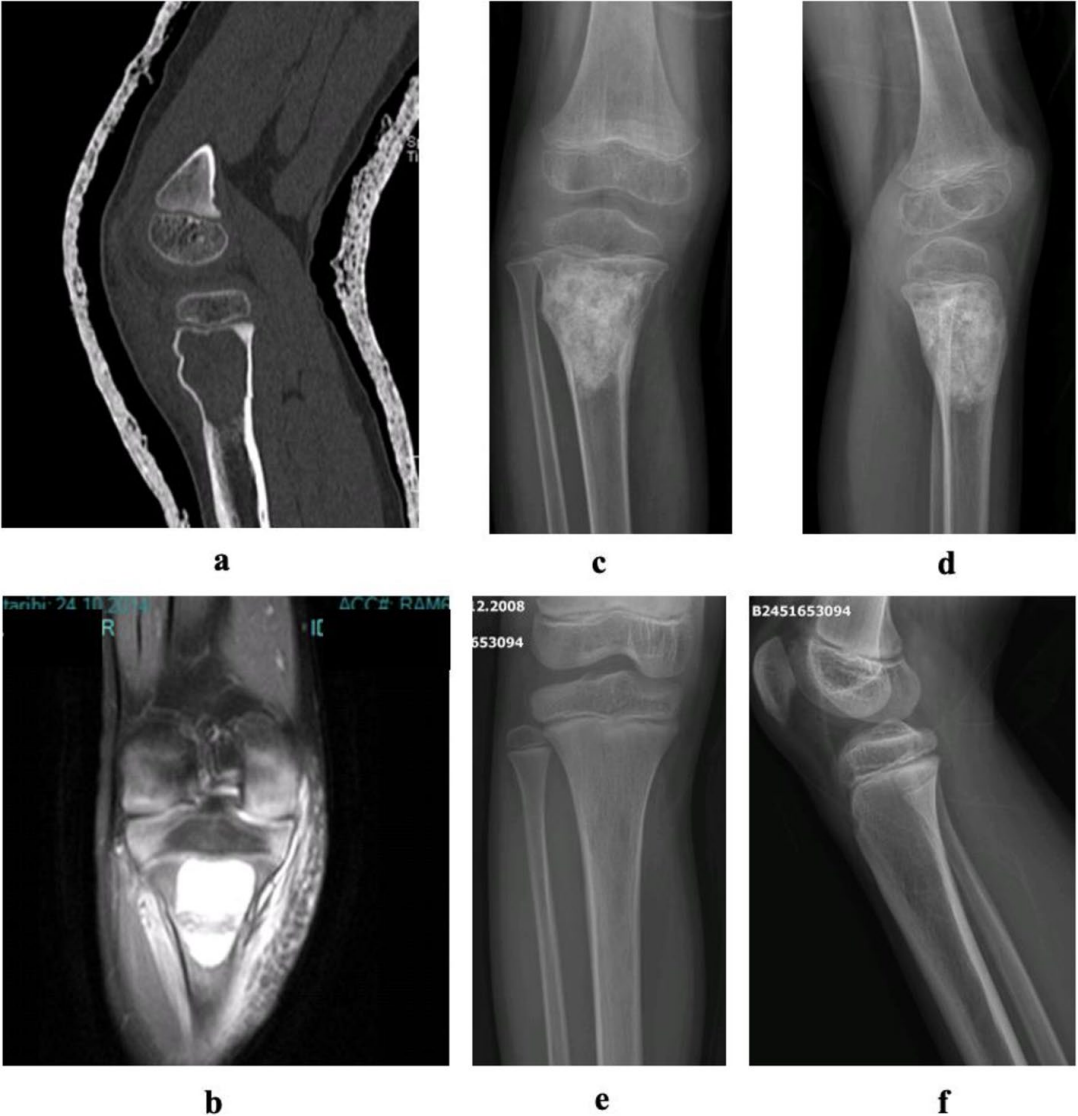
**a** CT section of the cyst. **b** MRI section of proximal tibial cyst. **c** Early postoperative AP radiograph. **d** Early postoperative lateral radiograph. **e** Postoperative 8th year AP Radiograph. **f** Postoperative 8th year lateral radiograph

**Fig. 5:**
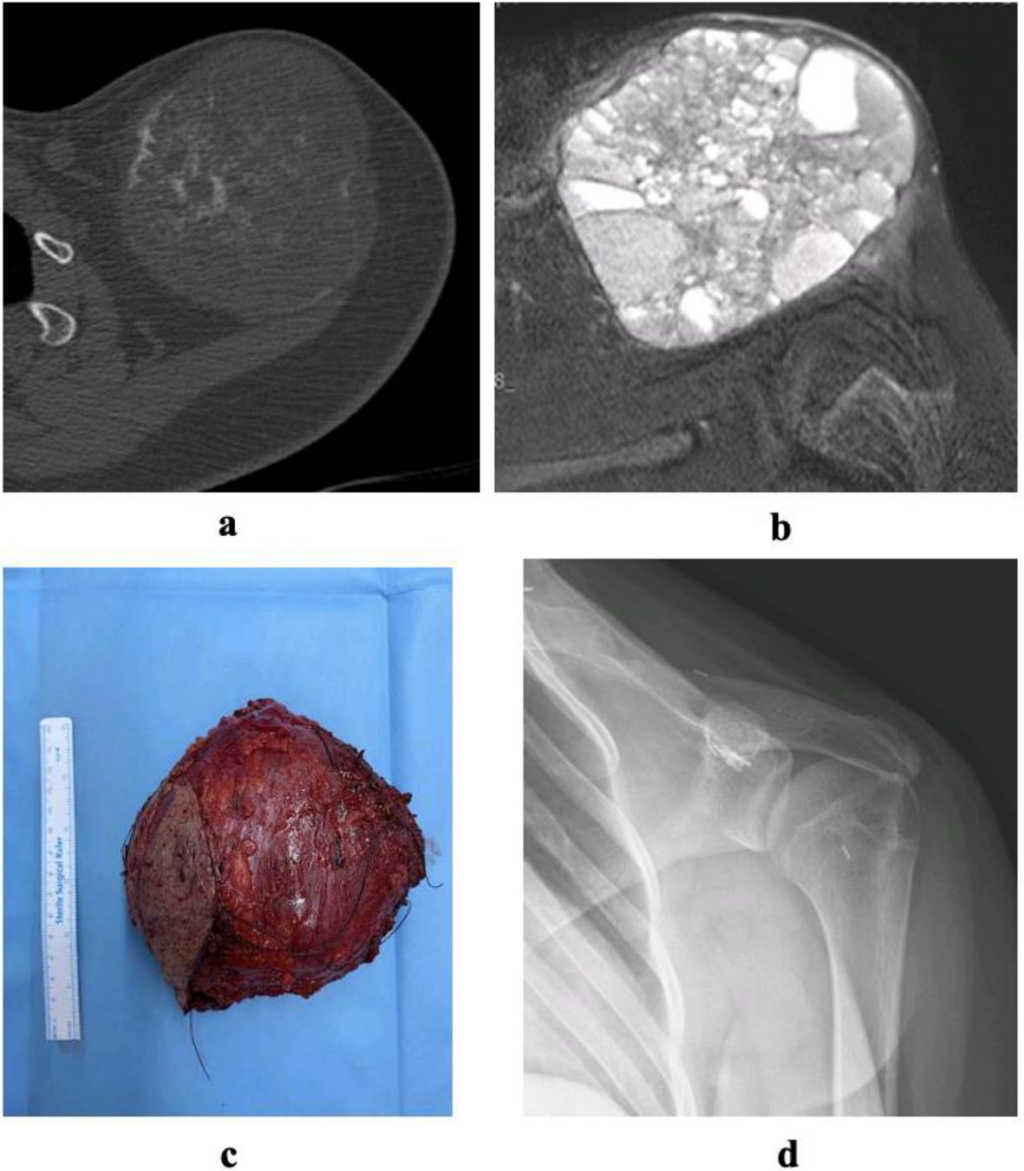
**a** CT axial section of aneurysmal bone cyst located in the clavicle. **b** MRI axial section of the cyst. **c** Peroperative resection material. **d** Postoperative radiograph after resection

Extended intralesional curettage was done in 96 patients (88.9%) while 12 (11.1%) underwent resection surgery. In curettage cases, 14 (13%) had no adjuvant, 56 (51.9%) used allograft, nine (8.3%) autograft and 17 (15.7%) bone cement as adjuvants. No additional local agents were used. Long-term images show an allograft-supported patient after curettage (Fig. 4). Resection cases (5 patients, 4.6%) used modular endoprosthesis or vascularized fibula and seven patients (6.5%) required no reconstruction. Images of a resected aneurysmal bone cyst in the clavicle are shown (Fig. 5).

No significant MSTS score difference was found among groups using bone cement, allograft, and autograft. However, a statistically significant difference was noted between resection-reconstruction and other groups (*p* < 0.001). At the end of the follow-up, 85 patients (78.7%) showed no complications. Relapse occurred in ten patients (9.3%) and 13 patients (12%) experienced various complications, including wound healing issues (7.4%), loss of reduction (1.9%) and neuropraxia, non-union and graft resorption (2.8%).

Patients with wound healing issues received dressing follow-up and those with loss of reduction were treated with titanium elastic nails and plates. Neuropraxia and graft resorption were managed conservatively with physical therapy. Patients with non-union underwent open reduction and internal fixation using iliac wing autograft after one year.

No significant correlations were found between complications and age (*p* = 0.874), gender (*p* = 0.780), tumor volume (*p* = 0.362) or radiological stage (*p* = 0.071). However, a lower age range was observed in male patients (*p* < 0.001). The MSTS score analysis was evaluated out of 100 points, with volume expressed in cm^3^. The numbers in the other variables indicate the number of patients. The MSTS score was significantly higher in the group without complications, as shown in Table 2.

**Table 2:** Complication analysis

	**Complication**			
	**No complication**	**Relapse**	**Other group**	***p***
Age	15 (11—24)	17 (8—28.5)	17 (12.5—25.5)	0.874*
Gender				
Female	37 (80.4)	3 (6.5)	6 (13.0)	0.780**
Male	48 (77.4)	7 (11.3)	7 (11.3)	
Volume	39.7 (11,670 −77,616)	56.5 (28,938 −248,475)	38.2 (11,193 −193,008)	0.362*
Stage				
Stage 2	72 (82.8)	6 (6.9)	9 (10.3)	0.071**
Stage 3	13 (61.9)	4 (19.0)	4 (19.0)	
Preoperative Biopsy				
No	36 (81.8)	3 (6.8)	5 (11.4)	0.831**
Yes	49 (76.6)	7 (10.9)	8 (12.5)	
Bone Type				
Long	78 (79.6)	10 (10.2)	10 (10.2)	0.147**
Others	7 (70.0)	0 (0.0)	3 (30.0)	
MSTS	100 (93.3—100.0)	93.3 (78.3—97.4)	96.6 (83.3—100.0)	0.003*

^*^Kruskal Wallis Test (median (Q1—Q3))

^**^Chi-Square Test (n(%))

Pathological fractures were significantly more common in the lower extremity (*p* = 0.028) with a notable association with a lower average age and higher incidence in male patients (*p* < 0.001; *p* = 0.041). Although patients with fractures had a numerically higher average mass volume, the statistical correlation with volume was not significant (*p* = 0.230).

## Discussion

Cysts are commonly observed in the first two decades of life with diagnostic features in radiological images [9, 13]. Preoperative (core needle biopsy) or frozen biopsy is often recommended for diagnosis [14, 15]. Studies in the literature have emphasized the significance of septa formation and the difference in fluid–fluid levels in confirming the diagnosis through preoperative MR and CT scans.

ABCs can be mistaken for various bone tumors in appearance and location [16, 17]. Differentiation from telangiectatic osteosarcoma characterized by malignant features and atypical cells is crucial [18]. Khal et al. also reported the clinical, radiological, and oncological outcomes of patients with low-grade central osteosarcoma, a rare subtype of osteosarcoma, as determined by diagnostic biopsy based on radiological features [19].

Literature supports investigating the relationship between mass volume and recurrence [3, 20]. Studies also explore factors like distance to the physis border and physis patency in recurrence risk [9, 21, 22]. Despite a numerically higher volume in recurrent cases, our study found no statistically significant relationship between cyst volume and recurrence (*p* = 0.362).

Although pathologic fractures were more common in lower extremities, they were proportionally higher in the upper extremities as well (*p* = 0.028). Younger age groups and males showed a higher prevalence of pathologic fractures (*p* = 0.041), consistent with literatüre [23].

In our study, no significant differences were found in age, gender, tumor volume, radiological stage and long-term complications. However, both recurrence and other complications were notably less common in patients undergoing extended intralesional curettage (*p* = 0.001). Furthermore, patients undergoing extended intralesional curettage had significantly higher MSTS scores compared to other surgical groups (*p* < 0.001).

MSTS scores were significantly higher in males and negatively correlated with age. Pediatric patients had notably higher MSTS scores (*p* < 0.001). Patients with a history of more than one operation and those evaluated in grade 3 (aggressive) based on the Campanacci classification had lower MSTS scores. The number of previous operations was statistically significant, but the radiologic classification's effect was not significant (*p* < 0.001; *p* = 0.088).

In related studies, recurrence rates varied (12–30%). Our study (108 patients) showed a lower recurrence at 9.7%. For example, Mankin et al. [20] reported 20% recurrence (150 patients), Vergel de Dios et al. [24] found 19% (153 patients), Ruiter et al. [25] reported 30.2% (105 patients) and Ramirez et al. [26] observed 27.5% recurrence in 29 pediatric cases.

In our long-term analysis, no significant differences were found in age, gender, tumor volume, radiological stage, bone type, preoperative biopsies or complications. However, the choice of surgical option (extended intralesional curettage or resection) was a significant factor in complications (*p* = 0.001). There are supporting studies in the literature on this subject [6, 9, 27].

Gender distribution's relationship with age was significant; males had a lower mean age (*p* < 0.001). Also, pathologic fractures were more common in lower extremities, with younger males having a higher prevalence (*p* = 0.028; *p* < 0.001; *p* = 0.041).

In the univariate analysis, significant variables (age, gender, upper extremity-lower extremity) were used in a multivariate model. For each one-unit increase in age, the likelihood of a pathological fracture increased by 1.1 times. In the lower extremity group, the probability of experiencing a pathological fracture is 3.61 times lower compared to the upper extremity group. In the univariate analysis, 54.3% of pathological fractures occurred in the lower extremity and 40% in the upper extremity. However, the percentage of fractures in the lower extremity group that were pathological fractures is 25.3%, whereas in the upper extremity group, this percentage is 53.8%. Hence, the likelihood of sustaining a fracture in the lower extremity is lower. In this context, a detailed analysis controlling for multiple independent variables (e.g., age, gender, limb location) on a single dependent variable (pathological fracture) is presented in Table 3.

**Table 3:** Multiple logistic regression analysis for pathologic fractures

	**Multivariate**			
**Variable**	**B**	**OR**	**%95 Confidence interval**	***P* value**
Age	−0.095	1.10	1.03—1.17	0.003
Gender	0.242	1.274	0.465—3.488	0.638
Extremity status		Reference		
Lower extremity	−1.283	3.61	1.29—10	0.014
Others	−0.493	0.611	0.082—4.537	0.630

From these evaluations, resection is not considered a primary treatment for ABCs. It's reserved for advanced cases with a history of surgeries and impaired soft tissue integrity. Resection patients in our practice had irregular follow-ups or inappropriate surgeries elsewhere. As a tertiary tumor center, we deemed resection necessary for this specific patient group. Evaluating their tumor volumes on admission, extended curettage was deemed unsuitable, leading us to opt for resection.

Curopsy, a biopsy technique aimed at cure, involves limited percutaneous curettage during biopsy, potentially resulting in a higher local recurrence rate compared to standard curettage [28]. Embolization is recognized as a beneficial adjunct in surgical therapy, particularly for surgically inaccessible sites like the spine or pelvis [29, 30]. While research is ongoing for various treatment modalities, bone marrow injection for primary aneurysmal bone cysts is not widely established in current medical literature, necessitating further research for local control confirmation.

Recent studies have explored the effectiveness of curettage and bone grafting for ABCs, a long-standing treatment [9, 31]. Attention has shifted to minimally invasive treatments, with promising outcomes from percutaneous injections of polidocanol or doxycycline showing low recurrence rates [32–34]. It's important to note that while some consider bone cement a chemical agent, studies suggest it doesn't generate high temperatures during application and doesn't cause bone necrosis [35, 36]. Larger studies are needed for conclusive evidence due to limited patient numbers and short follow-up periods.

Our study aims to comprehensively address the management and follow-up process of primary aneurysmal bone cysts in both pediatric and young adult age groups, with a focus on long-term treatment management. The strengths of this study include a large sample size, the application of advanced statistical methods, and the detailed comparative analysis between groups. These elements enhance the potential contribution of the findings to clinical decision-making processes and may serve as a valuable guide for improving treatment approaches.

## Limitations

Limitations of this retrospective study include the absence of a control group, variability in lesion localization and staging, and potential patient-dependent bias in MSTS scoring. Additionally, the study's retrospective nature may introduce selection bias, limiting causal inference. Despite the relatively long follow-up period, our ability to assess future outcomes, including recurrence rates, remains constrained. Due to the fact that not all patients were initially referred to or followed up at a single center, the lack of a standardized protocol across all cases prevented the development of uniform follow-up imaging and assessment procedures. Finally, while the sample size is adequate, the single-center design limits the generalizability of the findings to broader clinical settings.

Future studies should focus on multi-center, prospective designs to validate the findings from this single-center retrospective study, enabling broader generalization of the results. Additionally, further research is needed to establish standardized protocols for imaging and follow-up assessments, which could enhance the consistency and comparability of data across different clinical settings. Investigating long-term outcomes, such as recurrence rates and functional recovery over extended follow-up periods, would provide valuable insights into the durability and efficacy of current treatment modalities for aneurysmal bone cysts. Furthermore, future studies could explore the potential role of advanced molecular diagnostic techniques in improving the accuracy of diagnosis and treatment planning.

## Conclusions

Our manuscript discusses the effective application of extended curettage surgery with a high-speed burr and electrocautery for primary aneurysmal bone cysts at our tertiary tumor center. This approach shows promising long-term outcomes. In most cases of aneurysmal bone cysts, extended curettage using a high-speed burr and electrocautery, combined with grafting or cementing using polymethylmethacrylate and selective internal fixation, leads to low recurrence and complication rates over time. Patients should be aware that resection and reconstruction surgeries may result in functional bone loss and additional comorbidities, potentially limiting limb function. Treatment objectives should prioritize improving quality of life while minimizing functional loss, balancing benefits against potential drawbacks.

## Data Availability

No datasets were generated or analysed during the current study.

## Abbreviations

ABCs: Aneurysmal bone cysts
MSTS: Musculoskeletal Tumor Society
WHO: World Health Organization
CT: Computed tomography
MRI: Magnetic resonance imaging
